# Early response assessment and prediction of overall survival after peptide receptor radionuclide therapy

**DOI:** 10.1186/s40644-020-00335-w

**Published:** 2020-08-10

**Authors:** Daphne M. V. Huizing, Else A. Aalbersberg, Michelle W. J. Versleijen, Margot E. T. Tesselaar, Iris Walraven, Max J. Lahaye, Berlinda J. de Wit–van der Veen, Marcel P. M. Stokkel

**Affiliations:** 1 Department of Nuclear Medicine, Netherlands Cancer Institute, ENETS Center of Excellence, Amsterdam, The Netherlands; 2 Department of Medical Oncology, Netherlands Cancer Institute, ENETS Center of Excellence, Amsterdam, The Netherlands; 3 Department of Radiotherapy, Netherlands Cancer Institute, ENETS Center of Excellence, Amsterdam, The Netherlands; 4 Department of Radiology, Netherlands Cancer Institute, ENETS Center of Excellence, Amsterdam, The Netherlands

**Keywords:** PRRT, Therapy response, [^68^Ga]Ga-DOTA-TATE PET/CT, RECIST 1.1, Survival

## Abstract

**Background:**

Response after peptide receptor radionuclide therapy (PRRT) can be evaluated using anatomical imaging (CT/MRI), somatostatin receptor imaging ([^68^Ga]Ga-DOTA-TATE PET/CT), and serum Chromogranin-A (CgA). The aim of this retrospective study is to assess the role of these response evaluation methods and their predictive value for overall survival (OS).

**Methods:**

Imaging and CgA levels were acquired prior to start of PRRT, and 3 and 9 months after completion. Tumour size was measured on anatomical imaging and response was categorized according to RECIST 1.1 and Choi criteria. [^68^Ga]Ga-DOTA-TATE uptake was quantified in both target lesions depicted on anatomical imaging and separately identified PET target lesions, which were either followed over time or newly identified on each scan with PERCIST-based criteria. Response evaluation methods were compared with Cox regression analyses and Log Rank tests for association with OS.

**Results:**

A total of 44 patients were included, with median follow-up of 31 months (IQR 26–36 months) and median OS of 39 months (IQR 32mo-not reached)d. Progressive disease after 9 months (according to RECIST 1.1) was significantly associated with worse OS compared to stable disease [HR 9.04 (95% CI 2.10–38.85)], however not compared to patients with partial response. According to Choi criteria, progressive disease was also significantly associated with worse OS compared to stable disease [HR 6.10 (95% CI 1.38–27.05)] and compared to patients with partial response [HR 22.66 (95% CI 2.33–219.99)]. In some patients, new lesions were detected earlier with [^68^Ga]Ga-DOTA-TATE PET/CT than with anatomical imaging. After 3 months, new lesions on [^68^Ga]Ga-DOTA-TATE PET/CT which were not visible on anatomical imaging, were detected in 4/41 (10%) patients and in another 3/27 (11%) patients after 9 months. However, no associations between change in uptake on ^68^Ga-DOTA-TATE PET/CT or serum CgA measurements and OS was observed.

**Conclusions:**

Progression on anatomical imaging performed 9 months after PRRT is associated with worse OS compared to stable disease or partial response. Although new lesions were detected earlier with [^68^Ga]Ga-DOTA-TATE PET/CT than with anatomical imaging, [^68^Ga]Ga-DOTA-TATE uptake, and serum CgA after PRRT were not predictive for OS in this cohort with limited number of patients and follow-up time.

## Background

Peptide receptor radionuclide therapy (PRRT) for patients with metastatic or unresectable neuroendocrine tumours (NET) significantly increases progression free survival compared to conventional treatment and is expected to increase overall survival (OS) as well [1]. Mainly grade I and II NETs are treated with PRRT, since these tumours generally overexpress the PRRT target somatostatin receptor. Typically the treatment consists of four administrations of 7.4 GBq [^177^Lu]Lu-DOTA-TATE with 6–12 week intervals. Treatment response after PRRT can be determined using several different parameters: (1) anatomical changes measured on CT or MR imaging, (2) changes in uptake of [^68^Ga]Gallium-labeled somatostatin analogues (^68^Ga-SSA), or (3) change in tumour marker serum Chromogranin-A (CgA) levels [2]. Each of these three methods has its own advantages and drawbacks. Firstly, tumour size measurements on anatomical imaging is often performed according to the well-defined and reproducible Response Evaluation Criteria in Solid Tumours (RECIST 1.1) [3]. However, since NETs are slow-growing, there is a debate whether response or progression according to RECIST 1.1 is the right parameter [4]. To better differentiate the response of patients or tumour lesions, the more stringent Choi criteria for changes in tumour size (originally developed for slow-growing gastrointestinal stromal tumours) can be applied to NETs [5–7]. In addition, the presence of new bone lesions are often not observed with anatomical imaging, whereas [^68^Ga]Ga-DOTA-TATE PET/CT is able to visualize bone lesions from a certain diameter. Secondly, [^68^Ga]Ga-DOTA-TATE PET/CT has a high sensitivity and specificity for the detection of NETs [8]. Several PRRT studies have been performed to correlate baseline [^68^Ga]Ga-DOTA-TATE uptake to response (Positron Emission Tomography Response Criteria in Solid Tumors (PERCIST) and change on anatomical imaging) of the same lesion [9–11]. However, whether changes in [^68^Ga]Ga-DOTA-TATE uptake after PRRT on a patient level predicts survival is still unknown. Response monitoring using changes in [^68^Ga]Ga-DOTA-TATE uptake is also challenging since reduced tracer uptake could indicate a smaller number of somatostatin receptor (SSTRs) either due to disease progression (e.g., more SSTR-negative NET-cells or therapy response by a decline in the number of cells), or other parameters such as changes in perfusion. Thirdly, CgA levels are easily obtained, but have a moderate sensitivity and specificity in the follow-up setting for recurrence and/or progression [12]. The relationship between CgA and tumour load, however, remains debatable.

For clinicians and patients, the most important outcome concerning response assessment is the association with OS and determination of eligibility for treatment with subsequent cycles of PRRT. The aim of this study is to evaluate the role of anatomical- and receptor imaging and CgA level determination in PRRT response evaluation and their predictive value for OS.

## Materials and methods

### Patients and PRRT

Patients were considered suitable for PRRT in case of advanced well-differentiated NET grade 1–3 (confirmed by histopathology), with sufficient SSTR expression, as visualised by uptake on [^68^Ga]Ga-DOTA-TATE PET/CT. [^18^F]FDG PET/CT was performed to exclude patients for treatment with PRRT in case of presence of tumour lesions with increased metabolic activity without (increased) SSTR expression. MRI or contrast-enhanced CT, acquired in a different imaging session, were performed to assess changes in tumour size. Patients had to be in good condition according to WHO grade 0–1. Haematological parameters had to be above the following limits: Hb ≥5.5 mmol/L, leukocyte count ≥3.0 × 10^9^/L, neutrophil granulocyte count ≥1.0 × 10^9^/L, platelet count ≥75 × 10^9^/L. In addition, liver function and renal function should be adequate (total bilirubin ≤30 μmol/L, serum albumin ≥30 g/L, glomerular filtration rate (GFR) ≥50 ml/min/1.73m^2^). Renal outflow obstruction was excluded by [^99m^Tc]Technetium-MAG3 renal scintigraphy. Long-acting somatostatin analogues (SSAs) were discontinued four to 6 weeks before every treatment and short-acting SSAs for at least 24 h. A standard dosage of 7.4 GBq [^177^Lu]Lutetium-DOTA-TATE ([^177^Lu]Lu-DOTA-TATE) was administered four times at ten-week intervals. If deemed necessary due to subacute haematotoxicity, adjusted activity (3.7 or 5.5 GBq [^177^Lu]Lu-DOTA-TATE) was administered or next cycle was postponed until acceptable recovery of haematological parameters. All patients gave informed consent to use their data from routine clinical care for research purposes. Patients were selected consecutively, but were excluded from analysis in case of any other oncological treatments except for cold somatostatin analogues (SSA therapy) prior to the response assessment at 3 months after the last [^177^Lu]Lu-DOTA-TATE treatment, other tumours than NETs and in case of absence of follow-up scans in this time interval. If other therapies were applied between 3 and 9 months after PRRT, only the 9-month response assessment was excluded.

### Baseline and therapy evaluation

Baseline imaging included [^68^Ga]Ga-DOTA-TATE PET/CT within 6 months and morphological imaging (CT or MRI) within 2 months prior to start of PRRT according to clinical protocol. Laboratory parameters, including serum CgA levels were determined within 1 month prior to therapy start. Follow-up PET/CTs were performed in each patient at 3 months (accepted range 1–6 months) and 9 months (accepted range 6–12 months) after the fourth PRRT cycle according to local clinical protocol. Anatomical imaging and CgA level measurements were performed as close as possible to PET/CT imaging. PET/CT imaging was performed 45 min after the intravenous administration of 100 MBq of [^68^Ga]Ga-DOTA-TATE. Acquisition parameters included 3 min/bed from base of skull to mid-tights on Gemini ToF PET/CT systems (Philips, Best, The Netherlands) with 4x4x4mm voxel BLOB-OS-TF reconstruction. Low-dose CTs were additionally acquired for attenuation correction and anatomical correlation. SSA therapy was not withheld prior to [^68^Ga]Ga-DOTA-TATE imaging [13]. Contrast-enhanced (CE) CT imaging of thorax and/or abdomen was performed. If liver metastases were better visualized by MRI, contrast-enhanced MRI acquisitions of the liver only with mDixon, T2, and DWI sequences were performed.

### Image analysis

Target lesions were measured and classified on anatomical imaging, preferably CECT otherwise MRI, according to RECIST 1.1 criteria on baseline and 3 and 9 month follow-up scans [3]. The change in sum of diameters was additionally evaluated according to Choi criteria: increase ≥10% was classified as progression, decrease ≥10% as response, and in between as stable disease [5]. Measurements on anatomical imaging were performed in Vue PACS (Carestream, Rochester, NY) by an experienced radiologist (MJL) blinded for the clinical and PET/CT data.

[^68^Ga]Ga-DOTA-TATE uptake in RECIST 1.1 target lesions was measured and expressed in the standardized uptake values corrected for lean body mass (SUL_max_ and SUL_peak_) according to EANM guidelines [14]. Next, [^68^Ga]Ga-DOTA-TATE PET/CT scans were quantified using different methods based on PERCIST [15]. [^68^Ga]Ga-DOTA-TATE PET/CT target lesions were, independent of lesion size, identified using two methods: I) the most intense lesions at baseline were measurement on baseline and follow-up scans (‘Follow-up’) and II) the most intense lesions were defined on each scan individually (‘Independent’). PET target lesions were classified based on locations (liver, bone, soft tissue) with a maximum of two lesions per location and a total of five target lesions were identified using both methods ‘Follow-up’ and ‘Independent. In addition, also the single lesion with the highest uptake was noted. As a result, four categories were created: Follow-up5, Follow-up1, Independent5, and Independent1. The sum of SUL was used for comparison between baseline and follow-up scans in both Follow-up5 and Independent5. SUL measurements (DMH, EAA) were performed using the Image Computing Platform 3D Slicer (version 4.10).

In addition to tumour growth, the appearance of new lesions alone also indicates disease progression. Since cut-off values of tumour growth in NET are under discussion, the significance of new lesion detection was investigated separately by noting the absence or presence of new lesions on each scan compared to baseline.

### Data analysis

OS in months was determined from the start of PRRT until death from any cause or censured at last follow-up. Kaplan-Meier curves were plotted to compare median OS and inverse Kaplan-Meier was used to determine the follow-up time. Associations between imaging parameters, CgA levels and OS were evaluated using Cox survival analysis and Log Rank tests. Pearson’s correlation analysis was performed to assess the correlation between two evaluation methods. Statistical analysis was performed in SPSS (version 22, IBM, Armonk, NY) and PRISM (GraphPad, San Diego, CA).

## Results

**Fig. 1:**
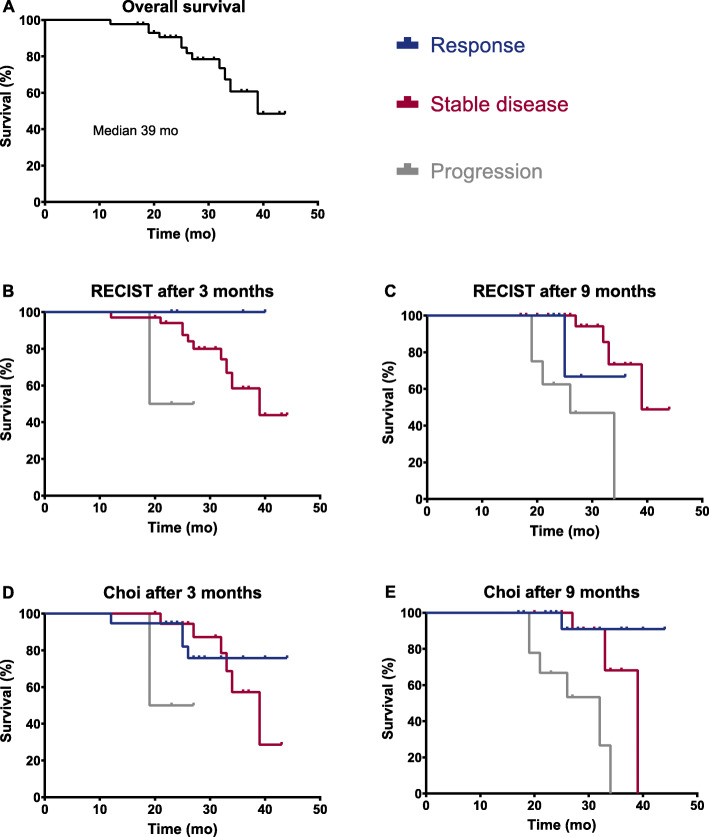
Kaplan-Meier curves of overall survival (**a**), and response according to RECIST 1.1 (**b-c**) and Choi (**d-e**)

A total of 44 patients were included in this retrospective study and all patients completed four cycles of PRRT. The average age at start of PRRT was 63.0 ± 9.6 years and 47.7% of patients were male. The median time between the baseline [^68^Ga]Ga-DOTATATE PET/CT and anatomical imaging modality and the first PRRT administration was 1.5 months [IQR 1-3mo] and 1 month [IQR 0-1mo], respectively. The median cumulative activity over four cycles was 29.7 GBq [^177^Lu]Lu-DOTA-TATE [IQR 29.3–29.9 GBq] and the median follow-up time was 31 months [IQR 26-36mo]. Median OS was 39 months [IQR 32mo-not reached] and 12 (27.3%) patients died during follow-up (Fig. 1a). CECT scans were performed in 41/44 patients, whereas 3/44 patients received baseline and follow-up MRI scans of the liver. [^68^Ga]Ga-DOTA-TATE PET/CT was performed in 41/44 patients (93.3%) after 3 months and in 27/44 patients (61.4%) after 9 months. All patient characteristics are shown in Table 1. In four patients only the 3-month time point was included due to radioembolisation treatment with [^166^Ho]Holmium-microspheres between 3 and 9 months after PRRT.

**Table 1:** Patient characteristics and number of scans performed

			Number (%)
Age		Years ± SD	63.0 ± 9.6
Gender		Female	23 (52.3%)
Primary tumour		Small intestine	28 (63.6%)
		Pancreas	11 (25.0%)
		Lung	3 (6.8%)
		Unknown	2 (4.5%)
Grade		1	26 (59.1%)
		2	16 (36.4%)
		3	2 (4.5%)
Anatomical imaging	Baseline	#	44 (100%)
	3 months	#	42 (95.5%)
		Time [range]	2 months [1–5]
	9 months	#	39 (88.5%)
		Time [range]	9 months [5–12]
[^68^Ga]Ga-DOTA-TATE PET/CT	Baseline	#	44 (100%)
	3 months	#	41 (93.3%)
		Time [range]	3 months [2, 3]
	9 months	#	27 (61.4%)
		Time [range]	9 months [6–12]
CgA	Baseline	#	44 (100%)
	3 months	#	42 (95%)
		Time [range]	2.5 months [1–6]
	9 months	#	39 (89%)
		Time [range]	9 months [6–12]

### Anatomical imaging assessment compared to OS

RECIST 1.1 measurement of 110 lesions was performed (62 liver lesions, 25 lymph nodes and 23 other soft tissue lesions). 81 and 66.7% of the patients showed stable disease according to RECIST 1.1 after 3 and 9 months respectively, whereas 45.2 and 33.3% of patients showed stable disease using Choi criteria (see Table 2). Median overall survival was not reached for all response groups using both RECIST 1.1 and Choi criteria, therefore the mean overall survival was estimated. Both RECIST 1.1 and Choi analysis after 3 months showed no association with OS (see Fig. 1 and supplementary materials).

**Table 2:** Response according to RECIST 1.1 and Choi criteria. Data is represented as (n (%))

		Response	Stable	Progression
3 months (*n* = 42)	RECIST 1.1	4 (9.5)	34 (81.0)	4 (9.5)
	Choi	19 (45.2)	19 (45.2)	4 (9.5)
9 months (*n* = 39)	RECIST 1.1	5 (12.8)	26 (66.7)	8 (20.5)
	Choi	17 (43.6)	13 (33.3)	9 (23.1)

Response groups according to RECIST 1.1 determined at 9 months showed significant differences in estimated mean OS: 39 months for patients with stable disease, 32 months for patients with response and 27 months for patients with progressive disease (Log Rank, *p* = 0.002). Progressive disease according to RECIST 1.1 at 9 months was significantly associated with worse OS compared to patients with stable disease [Cox regression, HR 9.04 (95% CI 2.10–38.85)].

Similar, response groups according to Choi criteria determined at 9 months showed significant differences in estimated mean OS: 37 months for patients with stable disease, 42 months for patients with response and 28 months for patients with progressive disease (Log Rank, *p* < 0.001). Progressive disease at 9 months according to Choi criteria was associated with worse OS compared to patients with stable disease [Cox regression, HR 6.10 (95% CI 1.38–27.05)] and compared to patients with response [Cox regression, HR 22.66 (95% CI 2.33–219.99)]. Figure 1 shows the survival curves for the total population as well as divided per response group according to RECIST 1.1 and Choi criteria after both 3 and 9 months.

Evaluation of the continuous variable of the cumulative size of target lesions also showed significant association between an increase in size and worse survival after 3 months [Cox regression, HR 1.041 (95% CI 1.015–1.068)] and after 9 months [Cox regression, HR 1.036 (95% CI 1.011–1.061)].

**Fig. 2:**
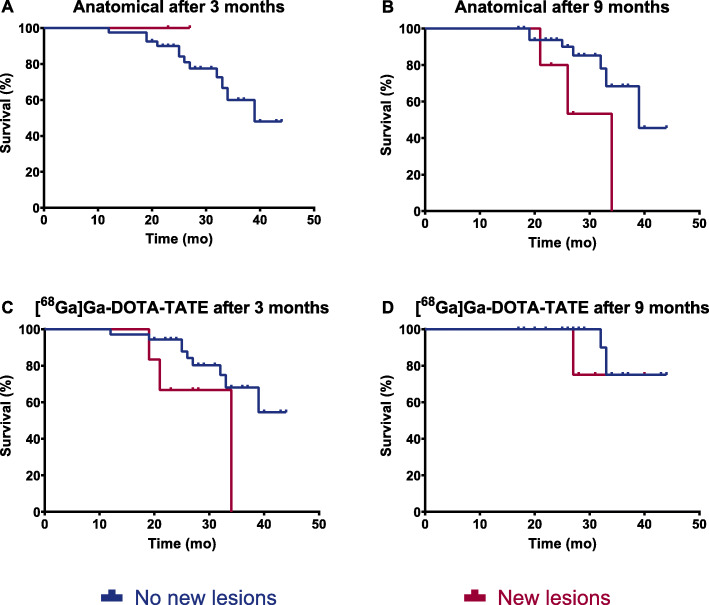
Kaplan-Meier curves of new lesions detected on CT/MRI (**a-b**) or [^68^Ga]Ga-DOTA-TATE PET/CT (**c-d**)

The appearance of new lesions in liver, bone and lung, detected on anatomical imaging with respect to baseline imaging, was reported in two patients (4.8%) after 3 months and in an additional three patients after 9 months, resulting in five patients (12.8%) with new lesions after 9 months. However, in this small group, no association between the presence of new lesions and OS was found (Fig. 2a-b).

### [^68^Ga]Ga-DOTA-TATE PET/CT analysis compared to OS

A total of 189 PET target lesions were segmented on baseline [^68^Ga]Ga-DOTA-TATE PET/CT (77 liver lesions, 35 bone lesions and 77 ‘other’ lesion). The lesions with the highest uptake were located primarily in the liver (61.4%), followed by ‘other’ locations (27.3%) and less often in the bone (11.4%). No associations between change in uptake on [^68^Ga]Ga-DOTA-TATE PET/CT using any of the four quantification methods and OS was observed (see supplementary materials).

New lesions in bone, liver and lung were detected in 6/41 patients (14.6%) after 3 months. An additional 3/26 patients (11.5%) showed new lesions after 9 months. Also these new lesions on [^68^Ga]Ga-DOTA-TATE PET/CT were not associated with OS, see Fig. 2c-d.

**Fig. 3:**
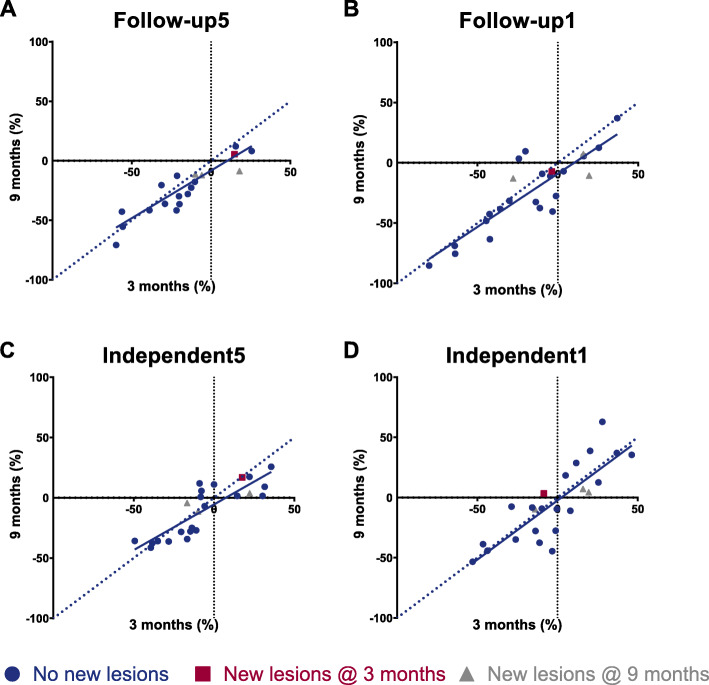
SUL_peak_ differences after 3 and 9 months with respect to baseline [^68^Ga]Ga-DOTA-TATE PET/CT of patients undergoing PET/CT at all three time points. Presence of new lesions on either scan 3 or 9 months is indicated with colours. Pearson correlation coefficients between both times points are (**a**) 0.888 with *p* < 0.001, (**b**) 0.862 with *p* < 0.001, (**c**) 0.846 with *p* < 0.001, and (**d**) 0.824 with *p* < 0.001

In Fig. 3, the correlation between SUL_peak_ measurements after 3 and 9 months is shown. There is a good correlation between image quantification after 3 and 9 months.

### Serum CgA evaluation compared to OS

The median CgA level at baseline was 739 μg/l [IQR 125–1746]. After 3 months, the median absolute CgA difference was − 87 μg/l [IQR − 341–16], and the percentage difference was − 15% [IQR -42–24]. At 9 months a median absolute CgA difference with respect to baseline of − 21 μg/l [IQR − 294–69] and a percentage difference of − 11% [IQR − 34–36] was observed. Neither the absolute differences in CgA nor the percentage difference after 3 or 9 months were associated with OS.

### Comparison between therapy evaluation methods

All new lesions observed on anatomical imaging were also detected on [^68^Ga]Ga-DOTA-TATE PET/CT, however not the other way around. After 3 months, 6 patients presented with new lesions on [^68^Ga]Ga-DOTA-TATE PET/CT, of whom only 2 patients presented with the same new lesions on anatomical imaging. In one patient a new liver lesion was detected on CT, however multiple liver lesions were already visible on [^68^Ga]Ga-DOTA-TATE PET/CT. On the other hand, no patients with new lesions on anatomical imaging alone were found. All results are shown in Table 3. After 9 months, an additional three patients showed new lesions on [^68^Ga]Ga-DOTA-TATE PET/CT but not on anatomical imaging. One patient showed new lesions on anatomical imaging, but a [^68^Ga]Ga-DOTA-TATE PET/CT was not available for comparison. Meanwhile, two patients that had new lesions after 3 months on [^68^Ga]Ga-DOTA-TATE PET/CT only showed new lesions on anatomical imaging after 9 months. In one patient different new lesions were detected (liver lesions on PET/CT and lymphadenopathy on CT), whereas in the other patient bone lesions were earlier observed on PET/CT than on CT.

**Table 3:** Presence or absence (N) of new lesions compared to baseline on each scan of all patients presenting with new lesions. n.a. = not acquired. ^1^Not visible on other imaging modality but within scan range. ^2^Not within scan range of other imaging modality. ^3^Visible on other imaging modality. ^4^No other imaging performed at this time point for comparison

Patient ID	PET/CT after 3 months ***n*** = 41	CT/MRI after 3 months ***n*** = 42	PET/CT after 9 months ***n*** = 27	CT/MRI after 9 months ***n*** = 39
9	Bone^2^ Liver^1^	N	n.a.	N
17	Bone^1^	N	n.a.	Other^4^
18	Bone^1^	N	n.a.	Bone^4^
24	Bone^3^	Bone^3^	n.a.	Bone^4^
29	Lung^3^	Lung^3^ Liver^1^	n.a.	Lung^4^ Liver^4^
35	Bone^1^	N	Bone^1^	N
5	N	N	Bone^1^	N
11	N	N	Liver^1^	N
22	N	N	Other^1^	N
10	N	N	n.a.	Other^4^

**Fig. 4:**
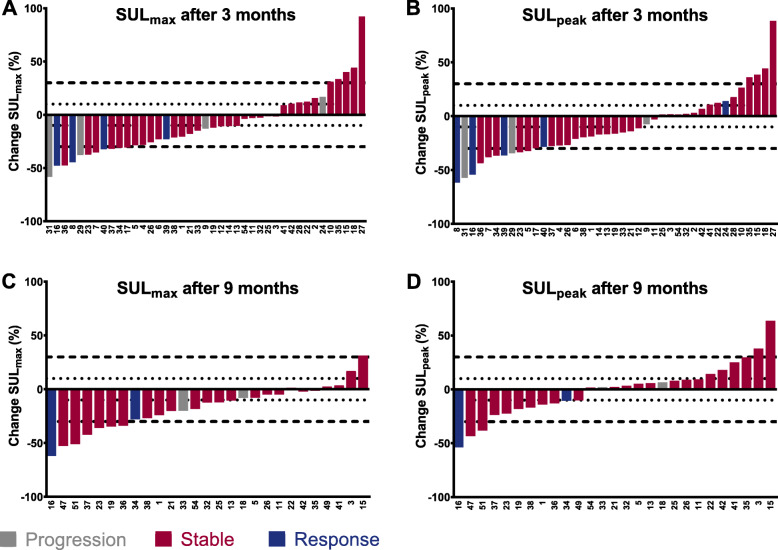
Waterfall plot of RECIST 1.1 outcome (response, stable disease or progression) and change in SUL-measurements of RECIST 1.1 target lesions for (**a**) SUL_max_ after 3 months, (**b**) SUL_peak_ after 3 months, (**c**) SUL_max_ after 9 months and (**d**) SUL_peak_ after 9 months

**Fig. 5:**
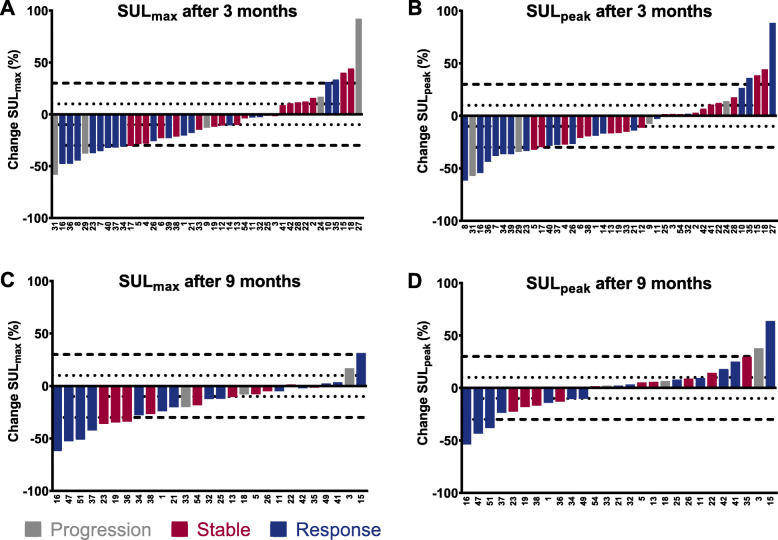
Waterfall plot of Choi results (response, stable disease, or progression) and change in SUL-measurements of the same target lesions after 3 months (**a-b**) and 9 months (**c-d**)

**Fig. 6:**
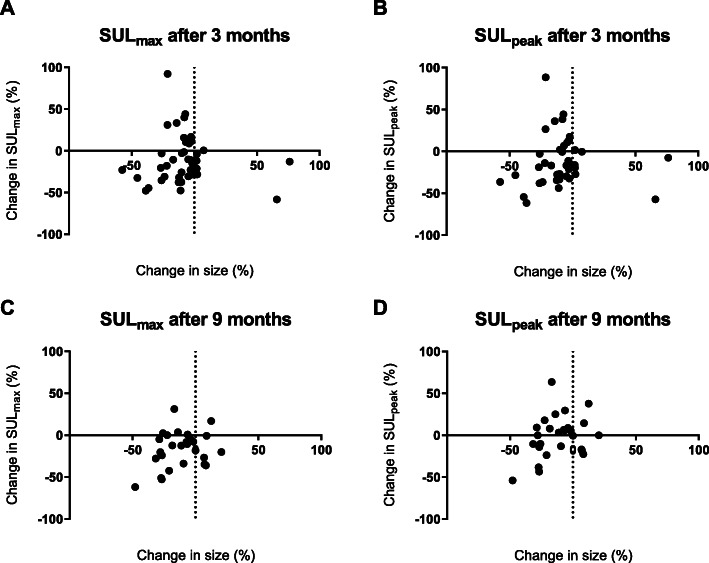
Comparison of percentage change in SUL and size of the same lesion. Pearson correlation coefficients between both time are (**a**) -0.030 with *p* = 0.852, (**b**) 0.039 with *p* = 0.810, (**c**) 0.304 with *p* = 0.123, and (**d**) 0.338 with *p* = p0.084

The response assessment methods were compared in different ways. At first, the change in size of the target lesions on anatomical imaging was compared to the [^68^Ga]Ga-DOTA-TATE uptake of the same RECIST 1.1 target lesions. The change in SUL in the RECIST 1.1 lesions with the corresponding RECIST 1.1 and Choi response category is shown in waterfall plots in Fig. 4 and Fig. 5, respectively. Although visually response evaluation according to Choi criteria shows improved concordance with SUL-measurements compared to RECIST 1.1 criteria, some patients classified with progressive disease still show a large decrease in SUL, whereas some patients with response still show an increase in SUL. No significant correlation was found between the percentage difference in diameter on anatomical imaging and the percentage difference in SUL_max_ or SUL_peak_ after 3 and 9 months (Fig. 6). Secondly, response based on the change in size of the target lesions on anatomical imaging was compared to SUL changes in separately identified PET target lesions quantified with all previously described methods. No significant association between response on anatomical imaging and change in [^68^Ga]Ga-DOTA-TATE uptake using all four quantification methods was observed. Thirdly, the percentage change in serum CgA level after 9 months and the response according to RECIST 1.1 at that time point were significantly different between the groups (*p* = 0.031). Patients with stable disease according to RECIST 1.1 had a decrease of CgA (median − 14.3%, IQR -30.0 - + 31.6%), responding patients also had a decrease in CgA (median − 42.3%, IQR -79.3 - -9.3%), whilst progressive patients showed an increase in CgA (median + 27.4%, IQR -11.7% - + 370.1%). There was no difference in CgA changes between Choi response groups.

## Discussion

Currently, different response assessment methods for anatomical imaging are applied in NET, mainly SWOG and/or RECIST (1.0 or 1.1) criteria [16]. In functional imaging, traditionally [^111^In]Indium-octreotide scans were visually assessed using the Krenning score, which compared tumour uptake with uptake in the liver and spleen/kidney [17]. However, with the introduction of SSA-labelled PET-tracers response evaluation of receptor imaging could be performed quantitatively. In current SSA-PET/CT research, tumour SUV_max_ is often used as a reference, but also the tumour-to-spleen and tumour-to-liver ratios are described [11, 18]. Previous studies used different selection criteria for target lesions, such as a single reference lesion with a diameter above 1.5 cm [9], a maximum of three lesions divided over four organs [18], or methods according to (modified)PERCIST [11]. The SUV_peak_, as recommended by PERCIST, is used rarely in current literature and was therefore taken into consideration here. In our study, progression on anatomical imaging determined using both RECIST 1.1 and Choi criteria after 9 months was associated with worse OS. Although new lesions were detected earlier with [^68^Ga]Ga-DOTA-TATE PET/CT than with anatomical imaging, changes in [^68^Ga]Ga-DOTA-TATE uptake, and serum CgA after PRRT were not predictive for OS.

Response on anatomical imaging, assessed by Choi criteria at 9 months, was associated with longer OS in this study. No association between response according to RECIST 1.1 at either time point and OS was observed, however this could be due to the low number of patients with response. The Cox survival analysis, however, was significantly different between patients with progressive disease compared to patients with stable disease after 9 months. This was also shown in other studies evaluating response within 1 year after the fourth cycle of PRRT treatment with [^90^Y]Yttrium-labelled and/or [^177^Lu]Lu-labelled compounds [19–21]. In addition, Vinjarmuri et al. observed that patients with both radiological, biochemical and clinical response had improved OS compared to the patients with partial response or stable disease [19]. In our study no cut-off values for change in [^68^Ga]Ga-DOTA-TATE uptake nor CgA levels were used to divide patients in response categories to allow for identification of certain threshold. The presented results, however, do not point to certain change [^68^Ga]Ga-DOTA-TATE uptake nor CgA levels that could be related with therapy response. In contrast to our study, Kong et al. did find associations between OS and response on anatomical imaging (CT), functional imaging ([^111^In]In-Octreotate SPECT, [^68^Ga]Ga-octreotate PET and [^18^F]FDG PET) and biochemical response using CgA levels [22]. Patients with response on all three response evaluation methods had significant improved OS. An important difference is that Kong et al. used the Krenning score for the receptor imaging modalities whereas methods inspired by PERCIST were applied in the current study. Although no ratios between normal tissue and tumour uptake were evaluated in this study, a decrease in tumour-to-spleen SUV ratio after PRRT is suggested to predict the time to progression according to RECIST 1.1 [18]. With respect to CgA, in this study the percentage change in serum CgA levels after 9 months was significantly different over all response groups according to RECIST 1.1. This is in line with the association between change in plasma CgA and tumour response according to RECIST 1.1 in 28 patients, observed by Kim et al. [23].

In this study, patients with progressive disease on anatomical imaging showed a significantly shorter OS compared to patients with stable disease for both RECIST 1.1 and Choi. It is important to notice that progressive disease is defined as either substantial growth of existing lesions and/or the appearance of new lesions. Therefore, the presence of new lesions alone was also investigated for association with OS. Although not statistically significant, again probably due to the small number of patients and short follow-up time, our results suggests that the presence of new lesions alone might be associated with a worse OS. Furthermore, new lesions were detected earlier or solely on [^68^Ga]Ga-DOTA-TATE PET/CT in 7/10 patients, whilst in 2/10 patients new lesions were detected simultaneously on both PET/CT and separate anatomical imaging. In 1/10 patients new lesions were detected on anatomical imaging only, but no [^68^Ga]Ga-DOTA-TATE PET/CT was available for comparison. This suggests that [^68^Ga]Ga-DOTA-TATE PET/CT might be the modality of choice for detection of new lesions that have arisen during or (shortly) after PRRT indicating therapy failure, which is relevant in decision making on (future) additional cycles of PRRT. To the best of our knowledge, the impact of progression due to the appearance of new lesions on survival has not yet been studied in NET. In non-small cell lung cancer patients, however, the new-lesion status on [^18^F]FDG PET/CT during erlotinib treatment was a potential surrogate biomarker for survival and treatment failure, being more informative than SUV measurements [24]. Similarly, increase in size of RECIST 1.1 target lesions was not predictive for OS, whilst the appearance of new lesions and progression of non-target lesions could predict OS in metastatic renal cell carcinoma patients [25].

The main limitations of this study are the small number of patients and limited follow-up time. As a result, the number of patients in our study presenting with new lesions is too small to provide any recommendations for patients presenting with new lesions after PRRT. Furthermore, most patients included in this study had grade I or grade II mid-gut NETs, of which the majority grade I which are slow growing tumours and require long follow-up times in order to observe progression of the disease. Also, the range of time between follow-up assessments is variable, which could affect the results. Although the protocol was to perform follow-up scans 3 and 9 months after PRRT, this is not always possible in routine clinical situations. Therefore, this study reflects clinical observations and these different types of progression after PRRT might warrant further investigation, as well as the role of [^18^F]FDG PET/CT.

PERCIST is developed and validated for [^18^F]FDG PET/CT imaging [15], but not for [^68^Ga]Ga-DOTA-TATE. Hence, other imaging analysis methods could be used for [^68^Ga]Ga-DOTA-TATE PET/CT assessment, for example the total tumour volume [26] or assessment of tracer distribution with texture analysis [27, 28]. However, PERCIST-like methods are easy to perform and are more likely to be adapted in the routine clinical practice. For that reason the focus in this study was on simple SUL-measurements, these have the highest chance for clinical implementation in our opinion. A disadvantage of our approach of measuring the most intense lesions on [^68^Ga]Ga-DOTA-TATE PET/CT is that probably low grade tumours are measured. Low grade (GI/GII) tumours are likely to show more somatostatin receptor expression than high grade tumours (GIII) and therefore more [^68^Ga]Ga-DOTA-TATE uptake [29]. It might be argued that these low-grade/high-uptake lesions respond better to PRRT, therefore measuring the lesions with the highest uptake might overestimate the response of the patient as a whole, however no evidence for this is available. Finally, clinical response assessment in patients with NETs is as important as imaging and laboratory derived parameters and PRRT has shown to improve quality of life [30]. Clinical response was however beyond the scope of the current study.

## Conclusion

Progression on anatomical imaging performed 9 months after PRRT is associated with worse OS compared to stable disease or partial response. Although new lesions were detected earlier with [^68^Ga]Ga-DOTA-TATE PET/CT than with anatomical imaging, [^68^Ga]Ga-DOTA-TATE uptake and serum CgA after PRRT were not predictive for OS in this cohort with limited number of patients and follow-up time.

## Supplementary information

**Additional file 1 Table S1.** Continuous variables and association with overall survival. **Table S2.** Categorized variables and association with overall survival. **Table S3.** Difference in CgA and uptake on [^68^Ga]Ga-DOTA-TATE PET/CT between response groups according to RECIST and Choi. **Table S4.** Detection of new lesions and association with overall survival.

## Data Availability

The datasets used in this manuscript are available from the corresponding author on reasonable request.

## Abbreviations

CE: Contrast-enhanced
CgA: Chromogranin-A
CI: Confidence interval
CT: Computed tomography
DWI: Diffusion weighted imaging
EANM: European Association of Nuclear Medicine
[^18^F]FDG: [^18^F]Fluorodeoxyglucose
[^68^Ga]Ga: ^68^Gallium
GBq: Gigabecquerel
GFR: Glomerular filtration rate
Hb: Haemoglobin
HR: Hazard ratio
[^177^Lu]Lu: ^177^Lutetium
MBq: Megebecquerel
MRI: Magnetic resonance imaging
NET: Neuroendocrine tumours
OS: Overall survival
PRRT: Peptide receptor radionuclide therapy
IQR: Interquartile range
RECIST: Response Evaluation Criteria in Solid Tumours
PERCIST: Positron Emission Tomography Response Criteria in Solid Tumors
PET: Positron emission tomography
SSA: Somatostatin analogue
SSTR: Somatostatin receptor
SUL: Standardized uptake value corrected for lean body mass
SUV: Standardized uptake value corrected for body weight
ToF: Time-of-flight
