# Supplementary material for: Early response assessment and prediction of overall survival after peptide receptor radionuclide therapy
Source: Cancer Imaging. 2020 Aug 10;20:57. doi: 10.1186/s40644-020-00335-w (PMC7418334; doi:10.1186/s40644-020-00335-w)
Supplement: Supplementary file 1 — Additional file 1 Table S1. Continuous variables and association with overall survival. Table S2. Categorized variables and association with overall survival. Table S3. Difference in CgA and uptake on [68Ga]Ga-DOTA-TATE PET/CT between response groups according to RECIST and Choi. Table S4. Detection of new lesions and association with overall survival. [file 40644_2020_335_MOESM1_ESM.docx]

**Supplementary materials**

**Table S1 Continuous variables and association with overall survival**

| Time point | Approach | HR (95% CI) | p-value |
| --- | --- | --- | --- |
| 3 months | CT size | 1.041 (1.015-1.068) | 0.002 |
|  | Follow-up5* | 1.007 (0.987-1.030) | 0.471 |
|  | Follow-up1* | 1.001 (0.983-1.018) | 0.939 |
|  | Independent5* | 1.008 (0.989-1.029) | 0.407 |
|  | Independent1* | 1.030 (0.974-1.090) | 0.300 |
|  | CgA absolute | 1.000 (1.000-1.000) | 0.015 |
|  | CgA percentage | 1.000 (1.000-1.000) | 0.009 |
| 9 months | CT size | 1.036 (1.011-1.061) | 0.004 |
|  | Follow-up5* | 1.020 (0.969-1.075) | 0.448 |
|  | Follow-up1* | 0.997 (0.966-1.030) | 0.861 |
|  | Independent5* | 1.004 (0.986-1.023) | 0.646 |
|  | Independent1* | 1.017 (0.983-1.052) | 0.325 |
|  | CgA absolute | 1.000 (1.000-1.000) | 0.009 |
|  | CgA percentage | 1.000 (1.000-1.000) | 0.009 |

*****[^68^Ga]Ga-DOTA-TATE PET/CT analysis using SUL_peak_

**Table S2 Categorized variables and association with overall survival**

| Time point | Approach | Category | HR (95% CI) | p-value |
| --- | --- | --- | --- | --- |
| 3 months | RECIST | Stable | 1 | 0.163 |
|  |  | Response | 0 | 0.984 |
|  |  | Progression | 4.943 (0.956-25.571) | 0.057 |
|  | Choi | Stable | 1 | 0.121 |
|  |  | Response | 0.780 (0.217-2.799) | 0.703 |
|  |  | Progression | 4.880 (0.867-27.460) | 0.072 |
| 9 months | RECIST | Stable | 1 | 0.011 |
|  |  | Response | 2.289 (0.236-22.185) | 0.475 |
|  |  | Progression | 9.042 (2.104-38.853) | 0.003 |
|  | Choi | Stable | 1 | 0.007 |
|  |  | Response | 0.269 (0.027-2.650) | 0.261 |
|  |  | Progression | 6.104 (1.377-27.054) | 0.017 |

**Table S3 Difference in CgA and uptake on [^68^Ga]Ga-DOTA-TATE PET/CT between response groups according to RECIST and Choi**

| Time point | Approach | p-value |
| --- | --- | --- |
| 3 months | RECIST vs. CgA absolute | 0.235 |
|  | RECIST vs. CgA percentage | 0.164 |
|  | Choi vs. CgA absolute | 0.216 |
|  | Choi vs. CgA percentage | 0.164 |
|  | RECIST vs. Follow-up5 | 0.006 |
|  | RECIST vs. Follow-up1 | 0.087 |
|  | RECIST vs. Independent5 | 0.045 |
|  | RECIST vs. Independent1 | 0.040 |
|  | Choi vs. Follow-up5 | 0.076 |
|  | Choi vs. Follow-up1 | 0.213 |
|  | Choi vs. Independent5 | 0.289 |
|  | Choi vs. Independent1 | 0.257 |
| 9 months | RECIST vs. CgA absolute | 0.031 |
|  | RECIST vs. CgA percentage | 0.078 |
|  | Choi vs. CgA absolute | 0.324 |
|  | Choi vs. CgA percentage | 0.421 |
|  | RECIST vs. Follow-up5 | 0.150 |
|  | RECIST vs. Follow-up1 | 0.842 |
|  | RECIST vs. Independent5 | 0.771 |
|  | RECIST vs. Independent1 | 0.979 |
|  | Choi vs. Follow-up5 | 0.522 |
|  | Choi vs. Follow-up1 | 0.929 |
|  | Choi vs. Independent5 | 0.384 |
|  | Choi vs. Independent1 | 0.254 |

**Table S4 Detection of new lesions and association with overall survival**

| Time point | Approach | HR (95% CI) | p-value |
| --- | --- | --- | --- |
| 3 months | Anatomical imaging | 0.047 (0.000-209941.54) | 0.695 |
|  | [^68­^Ga]Ga-DOTA-TATE PET/CT | 2.972 (0.780-11.325) | 0.111 |
| 9 months | Anatomical imaging | 3.809 (0.946-15.329) | 0.060 |
|  | [^68­^Ga]Ga-DOTA-TATE PET/CT | 2.801 (0.240-32.676) | 0.411 |
